# Preliminary feasibility study using a solution of synthetic enzymes to replace the natural enzymes in polyhemoglobin-catalase-superoxide dismutase-carbonic anhydrase: effect on warm ischemic hepatocyte cell culture

**DOI:** 10.3389/fbioe.2023.1231384

**Published:** 2023-08-07

**Authors:** M. Hoq, T. M. S. Chang

**Affiliations:** Artificial Cells and Organs Research Centre, Departments of Physiology, Medicine and Biomedical Engineering, Faculty of Medicine and Health Sciences, McGill University, Montreal, QC, Canada

**Keywords:** regenerative medicine, artificial cell, polyhemoglobin, synthetic enzyme, warm ischemia, oxygen carrier, antioxidant, CO_2_ carrier

## Abstract

This is a study on a simple solution of chemically prepared small chemical molecules of synthetic enzymes: catalase, superoxide dismutase, and carbonic anhydrase (CAT, SOD, and CA). We carried out a study to see if these synthetic enzymes can replace the natural enzymes (CAT, SOD, and CA) and avoid the need for the complicated cross-linking of natural enzymes to PolyHb to form PolyHb-CAT-SOD-CA. We compared the effect a solution of these three synthetic enzymes has on the viability of warm-ischemic hepatocytes that were exposed to nitrogen for 1 h at 37°C. PolyHb significantly increased the viability. The three synthetic enzymes themselves also significantly increased the viability. The use of both PolyHb and the three synthetic enzymes resulted in an additive effect in the recovery of viability. Increasing the concentration of the synthetic enzymes resulted in further increase in the effect due to the synthetic enzymes.

Implications: In addition to PolyHb, there are a number of other HBOC oxygen carriers. However, only Biopure’s HBOC product has received regulatory approval, but only in Russia and South Africa. None of the HBOCs has received regulatory approval by other countries. If regulatory agencies require HBOCs to have antioxidant or CO_2_ transport properties, all that is needed is to add or inject the solution of synthetic enzymes as a separate component.

## Introduction

This study is a part of the Frontiers special series on artificial cells in novel medical applications. The study on artificial cells (Chang, 2019) is a very large area, and we could only include 12 articles. Six of the 12 articles in this series are devoted to nanobiotechnology-based blood substitutes. These include experiences on the routine clinical uses in South Africa by Jahr, a bioengineering approach by Bulow, hemoglobin lipid vesicles by Sakai, use in preservation of transplant organs by Chan and Zhu, polyhemoglobin–catalase-superoxide dismutase–carbonic anhydrase (PolyHb-CAT-SOD-CA) by Bian and Chang, and the present article on the use of a solution of chemically prepared small enzyme molecules of CAT, SOD, and CA to replace the natural enzymes in PolyHb-CAT-SOD-CA. Since the other aspects have been discussed in detail in the other five articles, this paper will concentrate on the topic of synthetic enzymes.

Blood substitutes is a very broad area that has been reviewed in a recent open-access multiauthor book (Chang et al., 2022). One of the blood substitutes is polyhemoglobin, formed by the crosslinking of hemoglobin molecules. The use of gluteraldehyde to crosslink hemoglobin was first reported by Chang in 1971 (Chang, 1971). Since then, others have prepared a number of polyHbs using this basic glutaraldehyde method using bovine, human, pork, and cord blood Hb (Chang et al., 2022). Some of these have been developed and improved into products for clinical trials (Moore et al., 2009), and one has been approved for routine clinical use in South Africa and Russia (Mer et al., 2016; Chang, 2019). Laboratory research also includes the use of glutaraldehyde to prepare PolyHb-CAT-SOD-CA, which is an oxygen carrier with enhanced antioxidant function and CO2 transport function^5^. The last of the series is a brief research report on the use of synthetic CAT, SOD, and CA to replace the natural enzymes in PolyHb-CAT-SOD-CA. Thus, to have a valid comparison, we have selected our laboratory-made PolyHb and PolyHb-CAT-SOD-CA for comparison.

Natural enzymes have low stability, especially at body temperature. Nonhuman sources of natural enzymes are immunogenic and need complicated cross-linking with PolyHb to form PolyHb-CAT-SOD-CA. This study was performed to see if a simple solution of chemically prepared small chemical molecules of synthetic catalase, superoxide dismutase, and carbonic anhydrase (CAT, SOD, and CA) can replace the natural enzymes (CAT, SOD, and CA) in PolyHb-CAT-SOD-CA.

Synthetic enzymes are chemically prepared just to have the enzyme reaction sites of the large enzyme protein molecule (Bjerre et al., 2008). Zinc-based synthetic carbonic anhydrase is a novel approach (Liu et al., 2021). MnTmPyP is a commercially available manganese-containing metalloporphyrin synthetic superoxide dismutase (Zhu et al., 2016; Li et al., 2018; Gao et al., 2021). EUK-134 is a commercially available synthetic catalase (Dhanasekaran et al., 2018).

We perform this study to see if these synthetic enzymes can replace the natural enzymes we used for PolyHb-CAT-SOD-CA prepared and studied in this laboratory over the last 10 years. In order to compare the results, we have to use the same procedure of preparation for PolyHb-CAT-SOD-CA, including PolyHb (Table 1).

**TABLE 1:** Test solutions: (i) PolyHb and (ii) synthetic enzyme solution.

P_50_	Hb conc.	COP	Viscosity	MW	Hill coefficient, n50	MetHb content	K autoxidation (rate constant of autoxidation)	K NO (second-order rate constant of NO oxidation)
26.2 ± 0.2 mmHg	5 g/dL	20 mmHg	3 cP	>250 kDa	2.54 ± 0.03^19^	5.12% ± 1.67%	In the presence of superoxide and hydrogen peroxide, PolyHb shows oxidation of Hb (Fe2+) to metHb (Fe3+). This was prevented when PolyHb contains crosslinked antioxidant enzymes.	NO is important in maintaining the normal dilation of the microcirculation. Our present study studies the free suspension of hepatocytes. There is no microcirculation in the free suspension of hepatocytes, and thus, we did not follow this for the present study.

Organ and cell transplantation is dependent on the quality of donor organs and cells and their preservation before surgery. There have been promising results on the use of HBOCs for organ and cell pre-transplant preservation (Chang et al., 2021; Andrijevic et al., 2022; Chen et al., 2023). One of these has been approved in EU for organ preservation (Chen et al., 2023). Organs and cells vary greatly in their preservation requirements. The kidneys and limbs have lower requirements than the intestines or liver cells. Furthermore, the degree and duration of ischemia is also important. Again, for comparison, we have designed the present study based on our earlier studies of PolyHb and PolyHb-CAT-SOD-CA for hepatocytes in order to compare our results.

## Results

### Effects on regeneration of viability of warm ischemic human hepatocytes: Us

**FIGURE 1:**
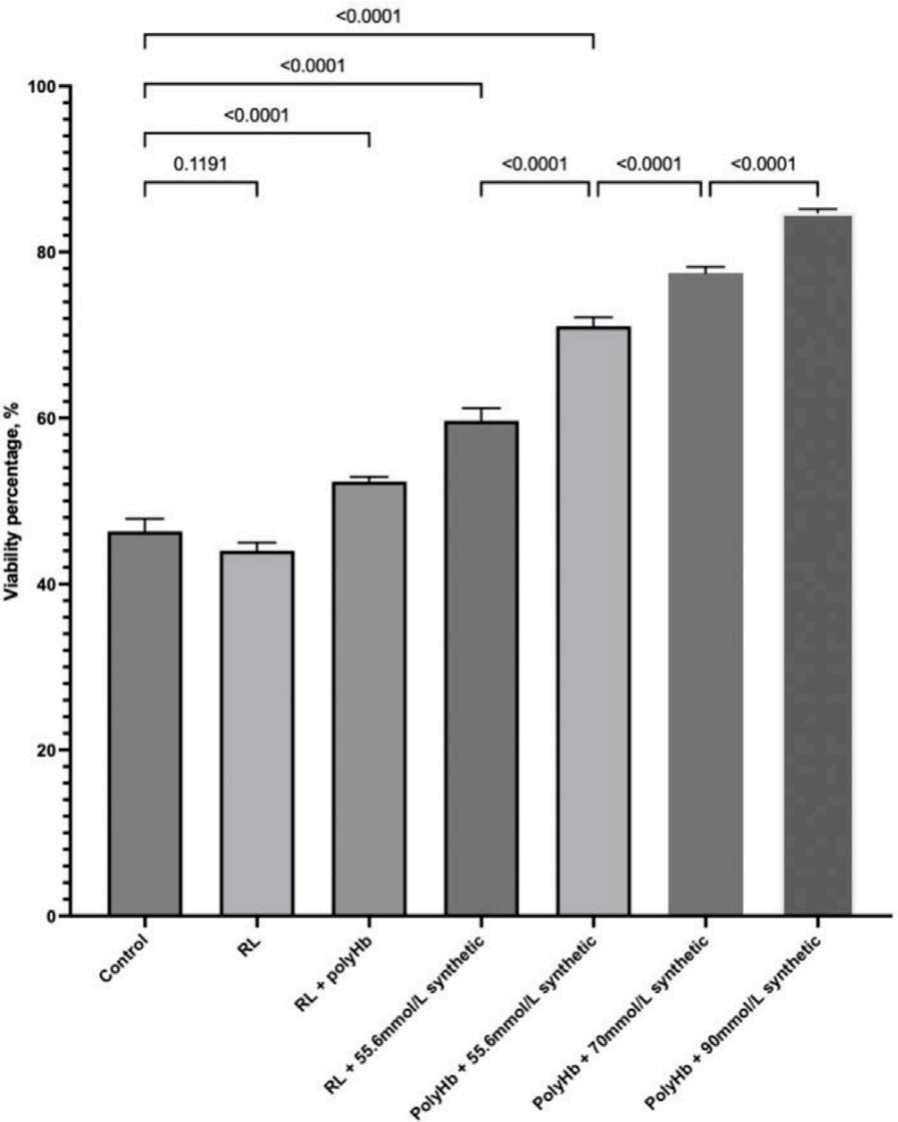
PolyHb significantly increased the viability. The three synthetic enzymes also significantly increased the viability. The use of both PolyHb and the three synthetic enzymes resulted in an additive effect in the recovery of viability. Increasing the concentration of the synthetic enzymes resulted in a further increase in the effect due to the synthetic enzymes.

We compared the effect of these synthetic enzymes on the viability of warm-ischemic hepatocytes that were exposed to nitrogen for 1 h at 37°C. PolyHb significantly increased the viability. The three synthetic enzymes also significantly increased the viability. The use of both PolyHb and the three synthetic enzymes resulted in an additive effect in the recovery of viability. Increasing the concentration of the synthetic enzymes resulted in further increase in the effect due to the synthetic enzymes (Figure 1).

### Stability analyses

**FIGURE 2:**
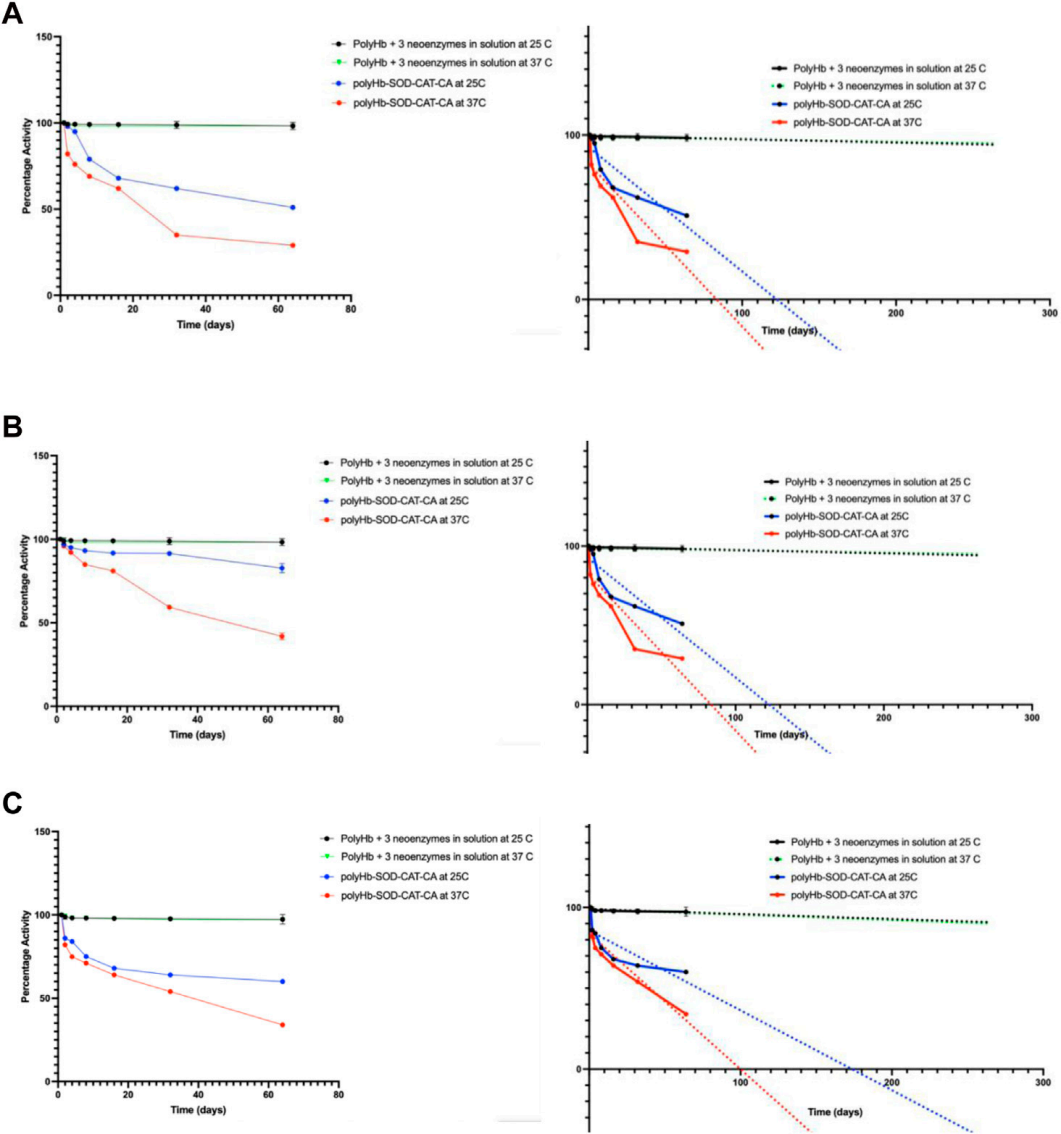
Comparing stability of neoenzymes and natural enzymes: **(A)** Carbonic anhydrase, **(B)** catalase, and **(C)** superoxide dismutase activities were analyzed over time at 25°C and 37°C for PolyHb + 3 synthetic enzymes in solution. These findings were then compared to our previous results on natural enzymes of PolyHb-SOD-CAT-CA (Bian et al., 2016). Copyright permission. The synthetic enzymes demonstrated significantly higher stability, nearly 100%, even when extrapolated to 260 days (see figures on the right). Results are presented as mean ± standard deviation (SD).

The three synthetic enzymes showed good stability of close to 100% even when extrapolated to 260 days at both room temperature and at 37°C body temperature (Figure 2). The natural enzymes, even in the more stable crosslinked form of PolyHb-CAT-SOD-CA, are by far less stable (Bian et al., 2016) (Figure 2).

### Enzyme activity assays

**FIGURE 3:**
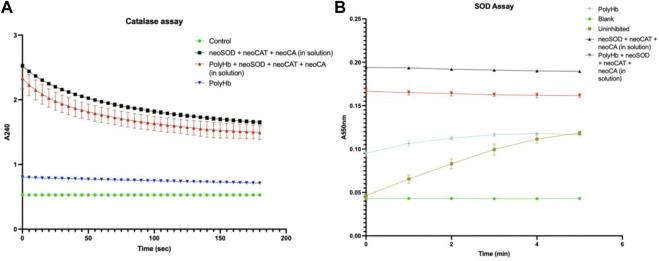
Enzyme activity assays of **(A)** neoCatalase and **(B)** neoSOD. The results are expressed as mean ± standard deviation (SD).

For carbonic anhydrase assay, the time taken to lower the pH of Tris-HCl from 8.3 to 6.3 is used to measure the W-A unit/ml of the solution. The CA activity for the PolyHb +three synthetic enzyme solution was significantly higher than for the control (no synthetic enzymes or PolyHb) and also significantly higher than for just PolyHb or just the three synthetic enzymes. For the control, it was 18.7 min; for the solution with neoenzymes, it was 4 min; for PolyHb alone, it was 1.1 min; and finally, for the novel solution containing PolyHb +three synthetic enzymes, it was 0.98 min. For the catalase assay, the time (in min) required to get a decrease of 0.05 absorbance unit was used to measure the activity in U/mL using the formula: U/mL = (3.45 x dilution factor)/(time x 0.1). The catalase activity of three synthetic enzymes in solution was 156,818 U/mL, while that of just PolyHb was 3450 U/mL, and that of PolyHb +three synthetic enzymes was 304,411 U/mL (Figure 3A). Lastly, for the superoxide dismutase assay, the enzyme activity was calculated in a coupled system, using xanthine and xanthine oxidase, where a unit of neoSOD that would inhibit the rate of reduction of cytochrome c by 50% is measured, and then, using the formula U/mL = (% inhibition) (DF)/(50%) (0.10), the activity was calculated. The SOD activity of PolyHb was 219.9 U/mL, that of just the three synthetic enzymes was 2,960.1 U/mL, and that of PolyHb +the three synthetic enzymes was 2,979.9 U/mL (Figure 3B). The enzyme activities of the synthetic enzymes in u/ml are shown in Table 2.

**TABLE 2:** Enzyme activities of the synthetic enzyme solution containing all the three synthetic enzymes in different concentrations.

Synthetic SOD activity U/mL	Synthetic CA activity W-A unit/mL	Synthetic CAT activity U/mL
1x (synthetic enzymes in the solution): 2,960	1x (synthetic enzymes in the solution): 360	1x (synthetic enzymes in the solution): 304,411
2x: 6,050	2x: 625	2x: 590,003
4x: 11,530	4x: 1,331	4x: 1,200,541
6x: 18,010	6x: 2,056	6x: 1,557,677

## Discussion

We are not comparing PolyHb prepared in this laboratory with other PolyHb products. Instead, we are performing this study to see if synthetic enzymes can replace the natural enzymes we used for PolyHb-CAT-SOD-CA prepared and studied in this laboratory over the last 10 years. In order to compare the results, we have to use the same procedure of preparation for PolyHb-CAT-SOD-CA including PolyHb.

This study is performed to see if a simple solution of chemically prepared small chemical molecules of synthetic enzymes catalase, superoxide dismutase, and carbonic anhydrase (CAT, SOD, and CA) can replace the natural enzymes (CAT, SOD, and CA). Natural enzymes have low stability, especially at body temperature. Nonhuman sources of natural enzymes are immunogenic and need complicated cross-linking with PolyHb to form PolyHb-CAT-SOD-CA.

The present study shows that the three synthetic enzymes showed good stability of close to 100% even when extrapolated to 260 days at both room temperature and at 37°C body temperature (Figure 2). The natural enzymes, even in the more stable crosslinked form of PolyHb-CAT-SOD-CA, are by far less stable (Figure 2).

We compared the effect of a solution of these three synthetic enzymes on the viability of warm-ischemic hepatocytes that were exposed to nitrogen for 1 hour at 37°C. PolyHb significantly increased the viability, most likely due to the supply of oxygen. The three synthetic enzymes also significantly increased the viability, most likely because of their antioxidant property and CO_2_ transport property. The use of both PolyHb and the three synthetic enzymes resulted in an additive effect in the recovery of viability from the supply of oxygen, antioxidant effect, and CO_2_ transport. Increasing the concentration of the synthetic enzymes (2x, 4x, and 6x) resulted in further increase in viability, most likely because of the severity of ischemic-reperfusion of the hepatocytes after 1 h in 37°C nitrogen incubation. This is similar to the use of natural enzymes, where increasing the enzyme by 2x, 4x, and 6x also increases the *in vivo* effect.

In summary, for this special case of hepatocytes after 1 h in 37°C nitrogen incubation, the simple solution of chemically prepared small chemical molecules of synthetic enzymes catalase, superoxide dismutase, and carbonic anhydrase (CAT, SOD, and CA) can replace the natural enzymes (CAT, SOD, and CA).

Future implications: There are a number of PolyHbs and HBOCs, including those that have been developed into products for clinical trials. However, only Biopure’s PolyHb product has received regulatory approval but only in Russia and South Africa. None of the PolyHbs has received regulatory approval by other countries. If regulatory agencies require PolyHb to have antioxidant property and CO2 transport property, all that is needed is to add or inject the solution of synthetic enzymes as a separate component—after further safety and efficacy study of the synthetic enzymes. This could reactivate the many HBOCs developed by many researchers and companies over the years.

## Methods

### Reagents and cell line

All chemical reagents including neoSOD, MnTmPyP, (CAT# 475872) neoCAT, and EUK-134, (CAT# SML0743) were purchased from Sigma-Aldrich, Canada. The human hepatocyte cell line (CAT# HMCPMS) and the thawing media (CM7000) used in this study were purchased from Thermo Fisher Scientific.

### Neo-carbonic anhydrase (neoCA) synthesis

NeoCA was synthesized according to a previous report^8^. Briefly, 0.51 g of zinc chloride and 1.72 g of L-histidine were added into a beaker containing 4.8 mL of glycerol. This was then stirred at 70**°**C until a homogenous liquid phase was formed, which resulted in the mixture changing color to light yellow. Finally, the mixture was then dried in a vacuum oven at 60**°**C for 2 h, which resulted in the ZnHisGly, or neoCA.

### Polyhemoglobin (PolyHb) preparation

PolyHb was prepared following the established protocol from this laboratory (Bian and Chang, 2015). Briefly, 4 mL of bovine hemoglobin was taken in a beaker (previously flushed with nitrogen gas and kept on ice throughout the process to prevent metHb formation). Then, 102 ul of 4 M sodium chloride along with 102 ul of 3 M potassium phosphate buffer were added to the beaker. Another 229 ul of 2 M lysine was added to it, and the mixture was shaken at 160 rpm at 4**°**C for 5 min. Without stopping the shaker, 162.4 ul of 0.5 M glutaraldehyde was added dropwise in four equal aliquots throughout a period of 15 min to start crosslinking. The beaker was kept shaking at 160 rpm overnight at 4**°**C. On the following day, 656 ul of 2 M lysine was added in two equal parts over 10 min and was shaken at 160 rpm for another hour at 4**°**C to stop crosslinking. The mixture was then centrifuged at 8,000 rpm at 4**°**C for 1 h to remove any precipitate. The supernatant was then purified using the Vivaspin 20 (CAT# GE28-9323–60) centrifugal filter at 2,500 g for 55 min at 4**°**C. This was exposed to vitamin C (0.05% w/v) for 24 h conc. Lastly, the retentate, containing just PolyHb, was collected and kept at −20**°**C for further use.

### Preparation of a novel solution containing polyhemoglobin plus neoSOD, neoCAT, and neoCA

Four solutions were prepared starting at a ratio of the oxygen carrier, PolyHb to neoSOD, neoCAT, and neoCA 1 g: 18 mg: 18 mg: 18 mg. Then, the synthetic enzymes were increased to 2x, 4x, and 6x.

### Enzymatic assays of the novel solution in comparison to PolyHb and a solution of just synthetic enzymes

The enzyme activities were measured following the procedures mentioned in a previous report (Mer et al., 2016). Briefly, carbonic anhydrase activity was determined by observing the time taken for the pH of 0.02 M Tris CO_2_ buffer to decrease from pH 8.3 to 6.3. For measuring the catalase activity, the rate at which hydrogen peroxide disappeared at a wavelength of UV 240 nm was monitored. Lastly, for measuring the activity of superoxide dismutase, the reduction of cytochrome C was used. There were four technical replicates used for these assays (*n* = 4).

### Stability analysis of the individual synthetic enzymes in the novel solution

The novel solution containing the synthetic enzymes and PolyHb was kept at both 37**°**C, body temperature, and at room temperature (RTP) for 2 months to check the stability of the synthetic enzymes. There were four technical replicates used for these stability analyses (*n* = 4).

### Human hepatocyte culture

Hepatocytes were thawed in warm CHRM^®^ medium, centrifuged at 100 x g for 10 min, and then mixed with plating medium (DMEM supplemented with 10% FBS and 1% penicillin/streptomycin) before being seeded (at a density of 2.4 × 10^5^ cells/mL) onto collagen-coated plates and incubated for 6 h at 37°C. The medium was then replaced with warm DMEM and incubated for 24 h. There were six technical replicates used for this study (*n* = 6).

### Regeneration of the viability of ischemic hepatocytes

The plates were transferred to a 37°C shaker with a constant flow of nitrogen gas to create an ischemic model. After 1 h, fresh DMEM was added, and the cells were treated with various combinations including the following:

1) Control hepatocytes treated with only fresh DMEM.
2) Hepatocytes treated with 20 ul Ringer’s Lactate (RL) and fresh DMEM.
3) Hepatocytes treated with 20 ul RL + 55.6 mmol/L synthetic enzymes in solution (neoSOD + neoCAT + neoCA) and fresh DMEM.
4) Hepatocytes treated with 0.626 mmol/L PolyHb +55.6 mmol/L synthetic enzymes in solution and fresh DMEM.
5) Hepatocytes treated with 0.626 mmol/L PolyHb +70 mmol/L synthetic enzymes in solution and fresh DMEM.
6) Hepatocytes treated with 0.626 mmol/L PolyHb +90 mmol/L synthetic enzymes in solution and fresh DMEM.

The plates were then placed back in the incubator for 24 h.

### Regenerative viability assay

Cells were washed twice with 2 mL PBS and treated with 4 mg/mL collagenase for 10 min. The reaction was stopped by adding 1.5 mL DMEM supplemented with FBS. The cells were then centrifuged at 1,500 rpm for 5 min and resuspended in 5 mL media. Cell count was determined by the trypan blue exclusion method, and cell viability was calculated using the following formula: percentage viability = (total no of cells - total no of cells in blue)/total no of cells x 100%

## Data analysis

The data were presented as mean ± standard deviation (SD). The differences among the control, RL, PolyHb, synthetic enzymes, and PolyHb + synthetic enzymes were assessed using one-way ANOVA. The significance level for all tests was set at 95%.

## Data Availability

The original contributions presented in the study are included in the article/supplementary material; further inquiries can be directed to the corresponding author.
